# Mr. Christie's Account of Vaccination in Ceylon

**Published:** 1809-02

**Authors:** Thomas Eaton

**Affiliations:** Halton


### *To the Editors of the Medical and Physical Journal.*

Gentlemen,

SHOULD you still admit Communications of any description relative to Vaccination, I send the enclosed, with the hope that you may think it acceptable to the Public. It shews the rapid progress Vaccine Inoculation is making in a distant quarter of the globe; where, I understand, Small-Pox formerly made dreadful ravages.

Mr. Christie's letter came to me through the medium of the worthy Vicar of Runcorn, whose brother, Captain Keyt, had it sent to him by Mr. Christie. Captain Keyt was lately in Ceylon with the 51st Regt.

I am, &c.

THOMAS EATON.

*Halton, Nov.* 25, 1808.

---

To the Editor of the Ceylon Government Gazette.

Sir,

I beg leave to enclose for publication, an Abstract of the number of persons vaccinated in Ceylon in 1807; which will shew the successful progress we have made in disseminating the practice of vaccination, throughout the island, during last year.

The number inoculated has been considerably greater than in any former year, particularly amongst the Malabar inhabitants of the Trincomallie and Jaffna districts, who in the first instance, seemed less disposed to adopt the practice than the Cingalese, in the south west part of the island; but a conviction of the perfect innocence of vaccination, and its preventive influnce against small-pox, is now very general amongst all ranks throughout the British possesions on Ceylon, though from the unfrequent occurrence of small-pox, the natives, in many places, shew more indifference and apathy about shielding themselves from that malady.

We do not, however, find any difficulty in keeping up the disease at the respective stations, but the vaccinators are now frequently obliged to visit the different villages, und urge the inhabitants to avail themselves of the benefits of inoculation; whereas, on the first introduction of the Cow-pox in 1802, when the Small-pox raged at Colombo, the natives of their own accord, flocked in crowds to to the inoculators, and expressed the greatest anxiety to be immediately vaccinated.

The small-pox was prevalent at Trincomallie in January last, and from thence found its way to Jaffna, but has since been banished from both places, by the beneficial influence of Vaccination, which has been very extensively practised in these districts during last year. The very successful propagation of the disease at these places may, I think, be attributed to the alarm created by the appearance of small-pox, conjoined with the beneficial effects of a Government Advertisement on the subject, circulated in the Malabar language, to which must be added the extreme assiduity of the Collectors, in promoting with their influence a diffusion of the practice, and the very meritorious exertions of the Medical Superintendants and Vaccinators in the discharge of their duty.

Previous to the introduction of cow-pox in 1802, the small-pox scarcely ever failed to visit us at Colombo during the prevalence of the south west monsoon, when the port was open, and generally carried off a great proportion of the inhabitants, but of late we have comparatively suffered very little from that disease. It is true, that since May 1805, we have had occasional cases of small-pox in the Pettah of this place, which, in some instances, there is reason to believe, was introduced from the Candian Country; but the contagion never spread as formerly, and is at present extinct, not only in the Colombo district, but throughout the whole of the British possessions on Ceylon, agreeably to the most certain information I have been enabled to procure, from the respective Vaccinators, who are directed to report on this subject.

From a Review of the Registers of Vaccination, I find that the total number of patients reported to me as having regularly passed through the disease, up to the end of 1806,—was 54,958, which, with 21,270, included in the Abstract for last year, will make a total of 76,828 persons; a large proportion of the limited population of these Settlements.

It would be absurd to expect that in such an extended practice, often conducted by persons not regularly educated to the profession of medicine, some failures and mistakes may not have taken place; but I can with truth affirm, that in the neighbourhood of Colombo, where small pox has most frequently occurred, I have been at great pains to trace to its source, any report prejudicial to vaccination, and to investigate the circumstances of every

K 4 case case of supposed failure; and in no one instance have I found that a person who had been vaccinated, and declared secure by the inoculator, ever afterwards had small-pox.

In a former letter I had occasion to mention, for the information of your Medical readers, the vaccine disease having been communicated to a boy affected with leprosy; and from a melancholy instance which has since occurred, it is certain that persons affected with that disease, in the most malignant form, are not exempt from the contagion of small-pox.

Clara de Silva, a woman, aged about 50 years, who had been confined in the Lepers Hospital since May 1775, with leprosy in the worst form, having been exposed to variolous contagion, sickened about the 1st of August, 1806, and died on the 11th of that month with confluent small-pox. — On the appearance of small-pox in the neighbourhood of the Lepers Hospital in May 1806, vaccination was practised amongst the patients of that Institution, but this old woman declared, she had had the small-pox when a child, and refused to be inoculated.

The fact may be useful, by shewing that no disease of the skin, however virulent, gives perfect security against small-pox; and that in the event of an epidemic contagion, no consideration of that nature ought to prevent us from attempting to shield the constitution against its influence, by vaccination.

*Abstract of the Number of Patients inoculated in the different Districts on Ceylon during the Year* 1807.

| Superintendants. | Districts. | Vaccinators. | Number. |
|---|---|---|---|
| A. High, Esq. | Caltura | F. W. De Hoedt | 2684 |
| | Columbo | H. W. Schimmelkettle | 1610 |
| | Negombo | M. Mack | 1153 |
| | Chelaw | J. H. Vansauden | 240 |
| | Calpentin | B. H. Toussaint | 799 |
| | Putlam | J. L. Janzen | 860 |
| J. A. Stutzer, Esq. | Manar | H. Mattheis | 1364 |
| | Jaffna | J. C. Keegel | 6083 |
| | Mullativo | J. C. De Hoedt | 247 |
| J. Bath, Esq. | Trincomalie | N. Claasz, & F. Van Sanden | 2016 |
| | Batticaloe | J. W. Seyp | 1070 |
| J. Adams, Esq. & M. Reynolds, Esq. | Hanbantotte | C. Hopman | 236 |
| | Tangalle | C. Hersse | 1556 |
| | Matura | J. W. Pietersz | 1277 |
| | Galle | J. Seybrands | 675 |

